# 2-Chloro-*N*-(3,5-dimethyl­phen­yl)acetamide

**DOI:** 10.1107/S160053680901304X

**Published:** 2009-04-18

**Authors:** B. Thimme Gowda, Sabine Foro, Hiromitsu Terao, Hartmut Fuess

**Affiliations:** a Department of Chemistry, Mangalore University, Mangalagangotri 574 199, Mangalore, India; b Institute of Materials Science, Darmstadt University of Technology, Petersenstrasse 23, D-64287 Darmstadt, Germany; c Faculty of Integrated Arts and Sciences, Tokushima University, Minamijosanjima-cho, Tokushima 770-8502, Japan

## Abstract

The conformation of the C=O bond in the structure of the title compound, C_10_H_12_ClNO, is *anti* to the N—H bond and to the C—Cl bond in the side chain in all four independent mol­ecules comprising the asymmetric unit. In the crystal, inter­molecular N—H⋯O hydrogen bonds link the mol­ecules into supra­molecular chains

## Related literature

For details of the preparation of the title compound, see: Shilpa & Gowda (2007 ▶). For related structures, see: Gowda *et al.* (2008**a* ▶,b* ▶,*c* ▶).
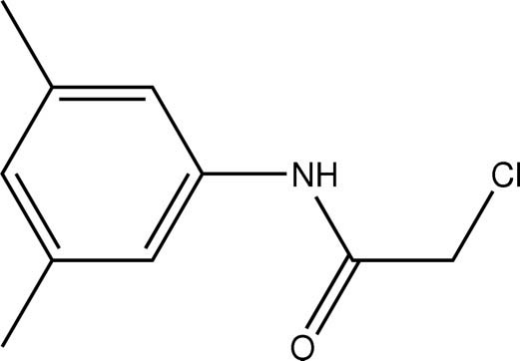


## Experimental

### 

#### Crystal data

C_10_H_12_ClNO
*M* *_r_* = 197.66
Orthorhombic, 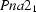

*a* = 25.9770 (1) Å
*b* = 9.7698 (4) Å
*c* = 16.0578 (7) Å
*V* = 4075.3 (3) Å^3^
*Z* = 16
Mo *K*α radiation
μ = 0.34 mm^−1^
*T* = 299 K
0.45 × 0.42 × 0.30 mm

#### Data collection

Oxford Diffraction Xcalibur diffractometer with a Sapphire CCD detector
Absorption correction: multi-scan (*CrysAlis RED*; Oxford Diffraction, 2007 ▶) *T*_min_ = 0.864, *T*_max_ = 0.906
27845 measured reflections
7450 independent reflections
4868 reflections with *I* > 2σ(*I*)
*R*_int_ = 0.021

#### Refinement

*R*[*F* ^2^ > 2σ(*F* ^2^)] = 0.050
*wR*(*F* ^2^) = 0.164
*S* = 1.04
7450 reflections
478 parameters
1 restraint
H-atom parameters constrained
Δρ_max_ = 0.53 e Å^−3^
Δρ_min_ = −0.20 e Å^−3^

### 

Data collection: *CrysAlis CCD* (Oxford Diffraction, 2004 ▶); cell refinement: *CrysAlis RED* (Oxford Diffraction, 2007 ▶); data reduction: *CrysAlis RED*; program(s) used to solve structure: *SHELXS97* (Sheldrick, 2008 ▶); program(s) used to refine structure: *SHELXL97* (Sheldrick, 2008 ▶); molecular graphics: *PLATON* (Spek, 2009 ▶); software used to prepare material for publication: *SHELXL97*.

## Supplementary Material

Crystal structure: contains datablocks I, global. DOI: 10.1107/S160053680901304X/tk2414sup1.cif

Structure factors: contains datablocks I. DOI: 10.1107/S160053680901304X/tk2414Isup2.hkl

Additional supplementary materials:  crystallographic information; 3D view; checkCIF report

## Figures and Tables

**Table 1:** Hydrogen-bond geometry (Å, °)

*D*—H⋯*A*	*D*—H	H⋯*A*	*D*⋯*A*	*D*—H⋯*A*
N1—H1*N*⋯O4^i^	0.86	2.14	2.983 (4)	168
N2—H2*N*⋯O3^ii^	0.86	2.12	2.975 (4)	171
N3—H3*N*⋯O2^iii^	0.86	2.15	3.000 (4)	170
N4—H4*N*⋯O1^iv^	0.86	2.13	2.987 (4)	172

Symmetry codes: (i) 
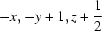
; (ii) 
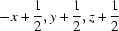
; (iii) 
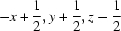
; (iv) 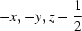.

**Fig. 1.**
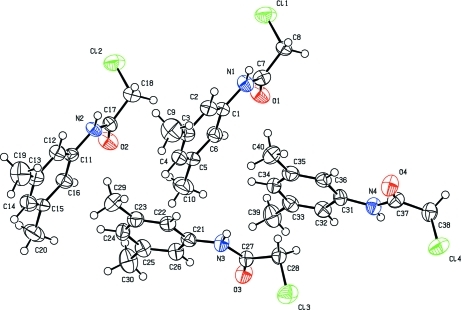
Molecular structure of (I), showing the atom labeling scheme and displacement ellipsoids drawn at the 50% probability level.

**Fig. 2.**
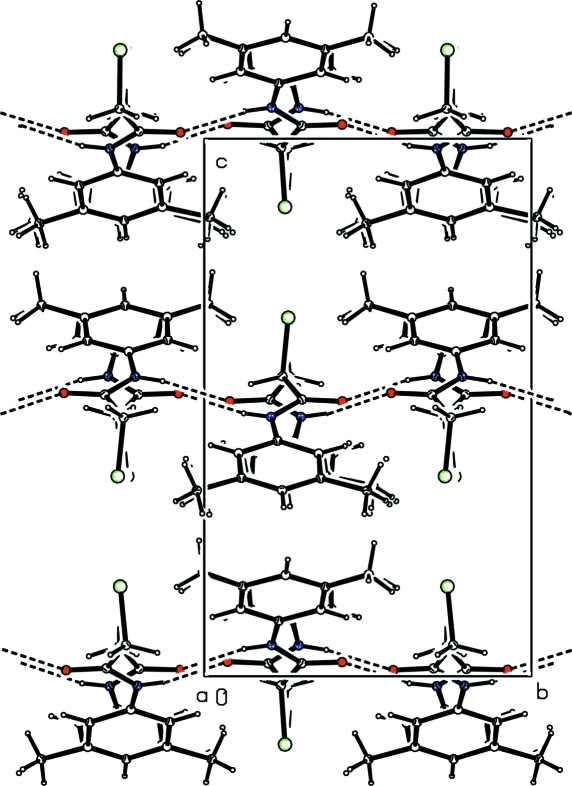
Molecular packing of (I) with hydrogen bonding shown as dashed lines.

## supplementary crystallographic information

### Comment

In the present work, as part of a study of substituent effects on the crystal structures of aromatic amides (Gowda *et al.*, 2008*a,b,c*), the structure of 2-chloro-*N*-(3,5-dimethylphenyl)acetamide (I) has been determined. The conformation of the C=O bond in (I) is *anti* to the N—H bond and to the C–Cl bond in the side chain (Fig. 1), in all the four independent molecules comprising the asymmetric unit. This is consistent with the *anti* conformation of the C=O bond to the N—H bond and to the side chain methylene H-atoms in 2-chloro-*N*- (2,4-dimethylphenyl)acetamide (Gowda *et al.*, 2008*a*), in 2-chloro-*N*-(3,5-dichlorophenyl)acetamide (Gowda *et al.*, 2008*b*), and in 2-chloro-*N*-(3-methylphenyl)acetamide (Gowda *et al.*, 2008*c*). The molecules in (I) are linked into infinite chains through intermolecular N—H···O hydrogen bonding (Table 1, Fig. 2). There are two independent supramolecular chains, one comprising O2- and O3- containing molecules, and the other comprising O1- and O4-containing molecules.

### Experimental

Compound (I) was prepared according to the literature method (Shilpa and Gowda, 2007). Single crystals were obtained from the slow evaporation of an ethanolic solution of (I).

### Refinement

The H atoms were positioned with idealized geometry using a riding model with C—H = 0.93–0.97 Å,  N—H = 0.86 Å, and were refined with isotropic displacement parameters set to 1.2 times of the *U*_eq_ of the parent atom. The structure was refined in the non-centrosymmetric space group Pna2_1_ with four independent molecules in the asymmetric unit. No evidence for higher symmetry was found but the structure was refined as a racemic twin.

### Crystal data

**Table:**

C_10_H_12_ClNO	*F*(000) = 1664
*M**_r_* = 197.66	*D*_x_ = 1.289 Mg m^−^^3^
Orthorhombic, *P**n**a*2_1_	Mo *K*α radiation, λ = 0.71073 Å
Hall symbol: P 2c -2n	Cell parameters from 7291 reflections
*a* = 25.9770 (1) Å	θ = 2.4–27.9°
*b* = 9.7698 (4) Å	µ = 0.34 mm^−^^1^
*c* = 16.0578 (7) Å	*T* = 299 K
*V* = 4075.3 (3) Å^3^	Prism, colourless
*Z* = 16	0.45 × 0.42 × 0.30 mm

### Data collection

**Table:**

Oxford Diffraction Xcalibur diffractometer with a Sapphire CCD detector	7450 independent reflections
Radiation source: fine-focus sealed tube	4868 reflections with *I* > 2σ(*I*)
graphite	*R*_int_ = 0.021
Rotation method data acquisition using ω and φ scans	θ_max_ = 25.4°, θ_min_ = 2.4°
Absorption correction: multi-scan (*CrysAlis RED*; Oxford Diffraction, 2007)	*h* = −22→31
*T*_min_ = 0.864, *T*_max_ = 0.906	*k* = −11→11
27845 measured reflections	*l* = −19→19

### Refinement

**Table:**

Refinement on *F*^2^	Primary atom site location: structure-invariant direct methods
Least-squares matrix: full	Secondary atom site location: difference Fourier map
*R*[*F*^2^ > 2σ(*F*^2^)] = 0.050	Hydrogen site location: inferred from neighbouring sites
*wR*(*F*^2^) = 0.164	H-atom parameters constrained
*S* = 1.04	*w* = 1/[σ^2^(*F*_o_^2^) + (0.0756*P*)^2^ + 2.0227*P*] where *P* = (*F*_o_^2^ + 2*F*_c_^2^)/3
7450 reflections	(Δ/σ)_max_ = 0.041
478 parameters	Δρ_max_ = 0.53 e Å^−^^3^
1 restraint	Δρ_min_ = −0.20 e Å^−^^3^

### Special details

**Table:**

Experimental. Absorption correction: CrysAlis RED, Oxford Diffraction Ltd., 2007 Empirical absorption correction using spherical harmonics, implemented in SCALE3 ABSPACK scaling algorithm.
Geometry. All esds (except the esd in the dihedral angle between two l.s. planes) are estimated using the full covariance matrix. The cell esds are taken into account individually in the estimation of esds in distances, angles and torsion angles; correlations between esds in cell parameters are only used when they are defined by crystal symmetry. An approximate (isotropic) treatment of cell esds is used for estimating esds involving l.s. planes.
Refinement. Refinement of F^2^ against ALL reflections. The weighted R-factor wR and goodness of fit S are based on F^2^, conventional R-factors R are based on F, with F set to zero for negative F^2^. The threshold expression of F^2^ > 2sigma(F^2^) is used only for calculating R-factors(gt) etc. and is not relevant to the choice of reflections for refinement. R-factors based on F^2^ are statistically about twice as large as those based on F, and R- factors based on ALL data will be even larger.

### Fractional atomic coordinates and isotropic or equivalent isotropic displacement parameters (Å^2^)

**Table:**

	*x*	*y*	*z*	*U*_iso_*/*U*_eq_	
Cl1	0.02134 (6)	0.25677 (12)	0.66479 (8)	0.0671 (5)	
O1	0.06503 (10)	0.0729 (2)	0.5120 (2)	0.0537 (7)	
N1	0.09129 (12)	0.2887 (3)	0.4815 (2)	0.0441 (8)	
H1N	0.0825	0.3731	0.4867	0.053*	
C1	0.13883 (16)	0.2643 (4)	0.4388 (3)	0.0380 (11)	
C2	0.16416 (16)	0.3814 (4)	0.4133 (3)	0.0442 (10)	
H2	0.1508	0.4672	0.4260	0.053*	
C3	0.21023 (17)	0.3698 (4)	0.3679 (3)	0.0474 (10)	
C4	0.22853 (18)	0.2446 (4)	0.3479 (4)	0.0517 (14)	
H4	0.2583	0.2380	0.3160	0.062*	
C5	0.20462 (16)	0.1281 (4)	0.3733 (3)	0.0490 (11)	
C6	0.15811 (15)	0.1369 (4)	0.4183 (3)	0.0430 (10)	
H6	0.1406	0.0579	0.4339	0.052*	
C7	0.05941 (15)	0.1988 (3)	0.5138 (3)	0.0437 (9)	
C8	0.01308 (17)	0.2590 (3)	0.5560 (3)	0.0366 (11)	
H8A	−0.0173	0.2066	0.5412	0.044*	
H8B	0.0081	0.3524	0.5372	0.044*	
C9	0.2371 (2)	0.5040 (5)	0.3424 (4)	0.0793 (17)	
H9A	0.2535	0.5438	0.3902	0.095*	
H9B	0.2120	0.5667	0.3206	0.095*	
H9C	0.2625	0.4850	0.3006	0.095*	
C10	0.22586 (19)	−0.0168 (5)	0.3544 (4)	0.0706 (15)	
H10A	0.2395	−0.0561	0.4046	0.085*	
H10B	0.2526	−0.0104	0.3134	0.085*	
H10C	0.1986	−0.0736	0.3336	0.085*	
Cl2	0.27157 (5)	0.24330 (13)	0.66707 (8)	0.0662 (5)	
O2	0.31648 (10)	0.0778 (2)	0.5114 (3)	0.0517 (8)	
N2	0.34182 (11)	0.2954 (3)	0.4823 (2)	0.0370 (8)	
H2N	0.3325	0.3792	0.4891	0.044*	
C11	0.38852 (15)	0.2759 (4)	0.4382 (3)	0.0332 (10)	
C12	0.41393 (15)	0.3947 (4)	0.4151 (3)	0.0412 (9)	
H12	0.4006	0.4796	0.4300	0.049*	
C13	0.45959 (17)	0.3868 (4)	0.3693 (3)	0.0487 (10)	
C14	0.47836 (19)	0.2619 (4)	0.3459 (4)	0.0531 (14)	
H14	0.5083	0.2569	0.3143	0.064*	
C15	0.45357 (15)	0.1443 (4)	0.3686 (3)	0.0429 (10)	
C16	0.40740 (15)	0.1502 (4)	0.4143 (3)	0.0422 (10)	
H16	0.3899	0.0704	0.4282	0.051*	
C17	0.31025 (13)	0.2014 (3)	0.5149 (3)	0.0354 (9)	
C18	0.26418 (17)	0.2609 (3)	0.5580 (3)	0.0389 (12)	
H18A	0.2333	0.2136	0.5400	0.047*	
H18B	0.2607	0.3569	0.5437	0.047*	
C19	0.48651 (18)	0.5228 (5)	0.3457 (4)	0.0703 (15)	
H19A	0.5033	0.5601	0.3939	0.084*	
H19B	0.4614	0.5867	0.3256	0.084*	
H19C	0.5116	0.5058	0.3030	0.084*	
C20	0.47403 (19)	0.0020 (5)	0.3456 (4)	0.0737 (16)	
H20A	0.4897	−0.0392	0.3936	0.088*	
H20B	0.4992	0.0104	0.3021	0.088*	
H20C	0.4461	−0.0543	0.3267	0.088*	
Cl3	0.14541 (5)	0.25313 (12)	−0.12686 (8)	0.0661 (5)	
O3	0.19027 (10)	0.0786 (2)	0.0264 (3)	0.0508 (8)	
N3	0.21661 (12)	0.2938 (3)	0.0580 (2)	0.0405 (8)	
H3N	0.2081	0.3780	0.0510	0.049*	
C21	0.26230 (16)	0.2724 (4)	0.1032 (3)	0.0392 (11)	
C22	0.29014 (16)	0.3888 (4)	0.1253 (3)	0.0417 (10)	
H22	0.2773	0.4742	0.1104	0.050*	
C23	0.33499 (16)	0.3822 (4)	0.1676 (3)	0.0460 (11)	
C24	0.35436 (17)	0.2513 (4)	0.1881 (4)	0.0460 (12)	
H24	0.3855	0.2445	0.2165	0.055*	
C25	0.32768 (16)	0.1304 (4)	0.1665 (3)	0.0485 (11)	
C26	0.28151 (15)	0.1437 (4)	0.1241 (3)	0.0428 (10)	
H26	0.2631	0.0658	0.1094	0.051*	
C27	0.18431 (13)	0.2016 (3)	0.0241 (3)	0.0347 (9)	
C28	0.13749 (17)	0.2653 (4)	−0.0172 (3)	0.0431 (13)	
H28A	0.1343	0.3605	−0.0008	0.052*	
H28B	0.1065	0.2173	−0.0001	0.052*	
C29	0.36440 (18)	0.5045 (5)	0.1930 (4)	0.0651 (14)	
H29A	0.3620	0.5157	0.2523	0.078*	
H29B	0.3505	0.5839	0.1659	0.078*	
H29C	0.3998	0.4935	0.1774	0.078*	
C30	0.34979 (18)	−0.0028 (5)	0.1894 (4)	0.0693 (15)	
H30A	0.3442	−0.0192	0.2477	0.083*	
H30B	0.3861	−0.0024	0.1782	0.083*	
H30C	0.3336	−0.0737	0.1575	0.083*	
Cl4	−0.10462 (5)	0.26428 (12)	−0.12784 (7)	0.0655 (4)	
O4	−0.05838 (10)	0.4286 (2)	0.0272 (2)	0.0571 (8)	
N4	−0.03326 (12)	0.2113 (3)	0.0574 (2)	0.0445 (8)	
H4N	−0.0418	0.1274	0.0491	0.053*	
C31	0.01203 (16)	0.2306 (4)	0.1042 (3)	0.0386 (11)	
C32	0.03858 (16)	0.1116 (3)	0.1258 (3)	0.0425 (10)	
H32	0.0248	0.0276	0.1100	0.051*	
C33	0.08350 (16)	0.1130 (4)	0.1687 (3)	0.0453 (10)	
C34	0.10421 (17)	0.2418 (4)	0.1895 (4)	0.0451 (12)	
H34	0.1352	0.2461	0.2185	0.054*	
C35	0.07887 (16)	0.3651 (4)	0.1673 (3)	0.0461 (11)	
C36	0.03352 (15)	0.3570 (4)	0.1245 (3)	0.0417 (9)	
H36	0.0168	0.4369	0.1086	0.050*	
C37	−0.06507 (14)	0.3041 (3)	0.0239 (3)	0.0431 (9)	
C38	−0.11124 (18)	0.2462 (4)	−0.0188 (4)	0.0482 (14)	
H38A	−0.1419	0.2938	−0.0002	0.058*	
H38B	−0.1149	0.1502	−0.0047	0.058*	
C39	0.11117 (17)	−0.0117 (5)	0.1926 (4)	0.0660 (14)	
H39A	0.0964	−0.0889	0.1644	0.079*	
H39B	0.1084	−0.0248	0.2517	0.079*	
H39C	0.1468	−0.0031	0.1775	0.079*	
C40	0.10289 (16)	0.4969 (4)	0.1907 (4)	0.0637 (14)	
H40A	0.1392	0.4930	0.1799	0.076*	
H40B	0.0972	0.5140	0.2488	0.076*	
H40C	0.0878	0.5694	0.1585	0.076*	

### Atomic displacement parameters (Å^2^)

**Table:**

	*U*^11^	*U*^22^	*U*^33^	*U*^12^	*U*^13^	*U*^23^
Cl1	0.0814 (11)	0.0752 (9)	0.0446 (10)	0.0129 (6)	0.0047 (8)	−0.0048 (5)
O1	0.0556 (16)	0.0297 (11)	0.076 (2)	0.0029 (10)	0.0151 (15)	−0.0013 (14)
N1	0.0427 (18)	0.0332 (15)	0.056 (2)	0.0030 (14)	0.0042 (16)	0.0020 (16)
C1	0.036 (2)	0.043 (2)	0.035 (3)	0.0020 (15)	0.004 (2)	−0.0045 (16)
C2	0.048 (2)	0.042 (2)	0.043 (3)	0.0026 (17)	0.005 (2)	0.0010 (18)
C3	0.043 (2)	0.060 (2)	0.039 (2)	−0.0051 (19)	0.008 (2)	0.011 (2)
C4	0.039 (3)	0.074 (3)	0.042 (3)	−0.0030 (19)	0.007 (2)	−0.007 (2)
C5	0.045 (3)	0.057 (2)	0.046 (3)	0.011 (2)	0.000 (2)	−0.016 (2)
C6	0.038 (2)	0.042 (2)	0.049 (2)	−0.0028 (16)	0.002 (2)	−0.0036 (19)
C7	0.058 (2)	0.0292 (17)	0.044 (2)	0.0024 (16)	−0.003 (2)	0.0017 (17)
C8	0.040 (3)	0.0286 (18)	0.041 (3)	0.0029 (14)	0.007 (2)	0.0000 (14)
C9	0.075 (3)	0.080 (3)	0.082 (4)	−0.028 (3)	0.015 (3)	0.018 (3)
C10	0.063 (3)	0.062 (3)	0.086 (4)	0.009 (2)	0.014 (3)	−0.020 (3)
Cl2	0.0789 (11)	0.0734 (8)	0.0463 (11)	0.0040 (6)	0.0062 (8)	−0.0082 (6)
O2	0.0581 (18)	0.0239 (12)	0.073 (2)	0.0009 (10)	0.0153 (16)	0.0023 (15)
N2	0.0368 (18)	0.0249 (14)	0.049 (2)	0.0074 (13)	0.0089 (16)	0.0004 (15)
C11	0.035 (2)	0.0337 (17)	0.030 (2)	−0.0054 (16)	0.0045 (19)	−0.0030 (19)
C12	0.043 (2)	0.0389 (19)	0.042 (2)	−0.0022 (16)	0.005 (2)	0.0033 (19)
C13	0.047 (2)	0.062 (2)	0.037 (2)	−0.009 (2)	0.006 (2)	0.012 (2)
C14	0.046 (3)	0.076 (3)	0.037 (3)	−0.001 (2)	0.010 (2)	0.006 (2)
C15	0.042 (2)	0.048 (2)	0.039 (2)	0.0080 (18)	0.005 (2)	−0.012 (2)
C16	0.045 (2)	0.0350 (19)	0.046 (2)	0.0002 (18)	0.008 (2)	−0.001 (2)
C17	0.033 (2)	0.0329 (18)	0.040 (2)	0.0041 (15)	0.0011 (19)	0.004 (2)
C18	0.048 (3)	0.0302 (19)	0.038 (3)	0.0001 (15)	0.008 (2)	0.0064 (16)
C19	0.064 (3)	0.064 (3)	0.082 (4)	−0.028 (2)	0.013 (3)	0.025 (3)
C20	0.059 (3)	0.078 (3)	0.084 (4)	0.012 (2)	0.020 (3)	−0.020 (3)
Cl3	0.0835 (11)	0.0721 (8)	0.0427 (9)	0.0096 (6)	−0.0094 (8)	0.0089 (5)
O3	0.0581 (18)	0.0260 (13)	0.068 (2)	−0.0002 (11)	−0.0152 (16)	0.0001 (15)
N3	0.043 (2)	0.0245 (14)	0.054 (2)	−0.0015 (14)	−0.0095 (17)	0.0031 (16)
C21	0.039 (3)	0.037 (2)	0.042 (3)	0.0033 (17)	0.004 (2)	−0.0036 (19)
C22	0.046 (2)	0.0318 (19)	0.048 (3)	0.0019 (16)	−0.001 (2)	−0.0038 (19)
C23	0.046 (2)	0.047 (2)	0.045 (3)	−0.0093 (18)	0.006 (2)	−0.006 (2)
C24	0.035 (3)	0.067 (3)	0.035 (3)	−0.0035 (18)	−0.003 (2)	0.0056 (16)
C25	0.042 (2)	0.052 (2)	0.051 (3)	0.0017 (19)	0.002 (2)	0.006 (2)
C26	0.042 (2)	0.036 (2)	0.051 (3)	−0.0012 (17)	−0.002 (2)	0.004 (2)
C27	0.036 (2)	0.0241 (17)	0.044 (2)	−0.0050 (15)	−0.0027 (19)	0.0051 (19)
C28	0.039 (3)	0.0334 (19)	0.057 (4)	0.0014 (15)	−0.006 (2)	−0.0026 (19)
C29	0.064 (3)	0.069 (3)	0.062 (3)	−0.007 (2)	−0.011 (3)	−0.016 (3)
C30	0.060 (3)	0.062 (3)	0.086 (4)	0.011 (2)	−0.008 (3)	0.028 (3)
Cl4	0.0838 (11)	0.0709 (8)	0.0418 (9)	−0.0003 (6)	−0.0079 (8)	−0.0071 (7)
O4	0.0615 (17)	0.0299 (12)	0.080 (2)	−0.0013 (11)	−0.0212 (16)	0.0020 (14)
N4	0.0435 (19)	0.0326 (15)	0.058 (2)	0.0063 (14)	−0.0085 (16)	0.0005 (16)
C31	0.031 (2)	0.0413 (19)	0.043 (3)	−0.0044 (16)	0.0005 (19)	0.0042 (18)
C32	0.048 (2)	0.0290 (17)	0.050 (2)	0.0006 (16)	0.002 (2)	0.0017 (17)
C33	0.045 (2)	0.046 (2)	0.046 (3)	0.0091 (17)	0.004 (2)	0.0091 (19)
C34	0.037 (3)	0.061 (3)	0.038 (3)	0.0042 (17)	−0.004 (2)	−0.0050 (17)
C35	0.048 (2)	0.048 (2)	0.043 (3)	−0.0026 (18)	0.007 (2)	−0.005 (2)
C36	0.043 (2)	0.0360 (19)	0.046 (2)	0.0040 (17)	0.002 (2)	−0.0038 (19)
C37	0.054 (2)	0.0293 (17)	0.046 (2)	0.0011 (16)	−0.004 (2)	−0.0017 (17)
C38	0.046 (3)	0.041 (2)	0.058 (4)	−0.0049 (15)	−0.007 (3)	0.0045 (18)
C39	0.055 (3)	0.067 (3)	0.076 (4)	0.012 (2)	−0.005 (3)	0.021 (3)
C40	0.059 (3)	0.055 (3)	0.077 (3)	−0.012 (2)	−0.006 (3)	−0.023 (2)

### Geometric parameters (Å, °)

**Table:**

Cl1—C8	1.761 (5)	Cl3—C28	1.778 (6)
O1—C7	1.239 (4)	O3—C27	1.212 (4)
N1—C7	1.314 (5)	N3—C27	1.346 (4)
N1—C1	1.433 (5)	N3—C21	1.407 (5)
N1—H1N	0.8600	N3—H3N	0.8600
C1—C6	1.381 (5)	C21—C26	1.394 (5)
C1—C2	1.382 (5)	C21—C22	1.393 (5)
C2—C3	1.405 (6)	C22—C23	1.350 (6)
C2—H2	0.9300	C22—H22	0.9300
C3—C4	1.351 (6)	C23—C24	1.413 (6)
C3—C9	1.540 (6)	C23—C29	1.476 (6)
C4—C5	1.360 (6)	C24—C25	1.413 (6)
C4—H4	0.9300	C24—H24	0.9300
C5—C6	1.410 (6)	C25—C26	1.385 (6)
C5—C10	1.549 (6)	C25—C30	1.469 (6)
C6—H6	0.9300	C26—H26	0.9300
C7—C8	1.501 (6)	C27—C28	1.519 (6)
C8—H8A	0.9700	C28—H28A	0.9700
C8—H8B	0.9700	C28—H28B	0.9700
C9—H9A	0.9600	C29—H29A	0.9600
C9—H9B	0.9600	C29—H29B	0.9600
C9—H9C	0.9600	C29—H29C	0.9600
C10—H10A	0.9600	C30—H30A	0.9600
C10—H10B	0.9600	C30—H30B	0.9600
C10—H10C	0.9600	C30—H30C	0.9600
Cl2—C18	1.770 (5)	Cl4—C38	1.768 (6)
O2—C17	1.220 (4)	O4—C37	1.230 (3)
N2—C17	1.338 (4)	N4—C37	1.339 (4)
N2—C11	1.418 (5)	N4—C31	1.409 (5)
N2—H2N	0.8600	N4—H4N	0.8600
C11—C16	1.377 (5)	C31—C36	1.393 (5)
C11—C12	1.386 (5)	C31—C32	1.396 (5)
C12—C13	1.397 (6)	C32—C33	1.356 (6)
C12—H12	0.9300	C32—H32	0.9300
C13—C14	1.366 (6)	C33—C34	1.409 (6)
C13—C19	1.549 (6)	C33—C39	1.466 (6)
C14—C15	1.367 (5)	C34—C35	1.418 (6)
C14—H14	0.9300	C34—H34	0.9300
C15—C16	1.407 (5)	C35—C36	1.367 (6)
C15—C20	1.534 (5)	C35—C40	1.479 (5)
C16—H16	0.9300	C36—H36	0.9300
C17—C18	1.500 (6)	C37—C38	1.493 (6)
C18—H18A	0.9700	C38—H38A	0.9700
C18—H18B	0.9700	C38—H38B	0.9700
C19—H19A	0.9600	C39—H39A	0.9600
C19—H19B	0.9600	C39—H39B	0.9600
C19—H19C	0.9600	C39—H39C	0.9600
C20—H20A	0.9600	C40—H40A	0.9600
C20—H20B	0.9600	C40—H40B	0.9600
C20—H20C	0.9600	C40—H40C	0.9600
			
C7—N1—C1	128.4 (3)	C27—N3—C21	129.5 (3)
C7—N1—H1N	115.8	C27—N3—H3N	115.3
C1—N1—H1N	115.8	C21—N3—H3N	115.3
C6—C1—C2	120.2 (4)	C26—C21—C22	119.3 (4)
C6—C1—N1	125.2 (4)	C26—C21—N3	124.1 (4)
C2—C1—N1	114.5 (3)	C22—C21—N3	116.6 (3)
C1—C2—C3	119.5 (4)	C23—C22—C21	122.5 (4)
C1—C2—H2	120.3	C23—C22—H22	118.8
C3—C2—H2	120.3	C21—C22—H22	118.8
C4—C3—C2	119.7 (4)	C22—C23—C24	117.9 (4)
C4—C3—C9	123.2 (4)	C22—C23—C29	123.2 (4)
C2—C3—C9	117.1 (4)	C24—C23—C29	119.0 (4)
C3—C4—C5	121.8 (5)	C25—C24—C23	121.6 (4)
C3—C4—H4	119.1	C25—C24—H24	119.2
C5—C4—H4	119.1	C23—C24—H24	119.2
C4—C5—C6	119.6 (4)	C26—C25—C24	117.8 (4)
C4—C5—C10	122.9 (4)	C26—C25—C30	123.0 (4)
C6—C5—C10	117.5 (4)	C24—C25—C30	119.1 (4)
C1—C6—C5	119.2 (4)	C25—C26—C21	120.9 (4)
C1—C6—H6	120.4	C25—C26—H26	119.6
C5—C6—H6	120.4	C21—C26—H26	119.6
O1—C7—N1	125.5 (4)	O3—C27—N3	124.8 (4)
O1—C7—C8	119.6 (3)	O3—C27—C28	121.5 (3)
N1—C7—C8	114.9 (3)	N3—C27—C28	113.7 (3)
C7—C8—Cl1	110.2 (3)	C27—C28—Cl3	108.2 (3)
C7—C8—H8A	109.6	C27—C28—H28A	110.1
Cl1—C8—H8A	109.6	Cl3—C28—H28A	110.1
C7—C8—H8B	109.6	C27—C28—H28B	110.1
Cl1—C8—H8B	109.6	Cl3—C28—H28B	110.1
H8A—C8—H8B	108.1	H28A—C28—H28B	108.4
C3—C9—H9A	109.5	C23—C29—H29A	109.5
C3—C9—H9B	109.5	C23—C29—H29B	109.5
H9A—C9—H9B	109.5	H29A—C29—H29B	109.5
C3—C9—H9C	109.5	C23—C29—H29C	109.5
H9A—C9—H9C	109.5	H29A—C29—H29C	109.5
H9B—C9—H9C	109.5	H29B—C29—H29C	109.5
C5—C10—H10A	109.5	C25—C30—H30A	109.5
C5—C10—H10B	109.5	C25—C30—H30B	109.5
H10A—C10—H10B	109.5	H30A—C30—H30B	109.5
C5—C10—H10C	109.5	C25—C30—H30C	109.5
H10A—C10—H10C	109.5	H30A—C30—H30C	109.5
H10B—C10—H10C	109.5	H30B—C30—H30C	109.5
C17—N2—C11	128.9 (3)	C37—N4—C31	129.7 (3)
C17—N2—H2N	115.6	C37—N4—H4N	115.1
C11—N2—H2N	115.6	C31—N4—H4N	115.1
C16—C11—C12	120.2 (4)	C36—C31—C32	118.8 (4)
C16—C11—N2	124.3 (3)	C36—C31—N4	125.3 (4)
C12—C11—N2	115.4 (3)	C32—C31—N4	115.7 (3)
C11—C12—C13	119.9 (4)	C33—C32—C31	122.9 (4)
C11—C12—H12	120.1	C33—C32—H32	118.5
C13—C12—H12	120.1	C31—C32—H32	118.5
C14—C13—C12	119.8 (4)	C32—C33—C34	117.3 (4)
C14—C13—C19	122.5 (4)	C32—C33—C39	123.2 (4)
C12—C13—C19	117.7 (4)	C34—C33—C39	119.5 (4)
C13—C14—C15	120.6 (4)	C33—C34—C35	121.5 (4)
C13—C14—H14	119.7	C33—C34—H34	119.3
C15—C14—H14	119.7	C35—C34—H34	119.3
C14—C15—C16	120.4 (4)	C36—C35—C34	118.5 (4)
C14—C15—C20	122.3 (4)	C36—C35—C40	122.8 (4)
C16—C15—C20	117.3 (4)	C34—C35—C40	118.7 (4)
C11—C16—C15	119.0 (3)	C35—C36—C31	121.0 (4)
C11—C16—H16	120.5	C35—C36—H36	119.5
C15—C16—H16	120.5	C31—C36—H36	119.5
O2—C17—N2	125.5 (4)	O4—C37—N4	124.4 (4)
O2—C17—C18	120.7 (3)	O4—C37—C38	120.5 (3)
N2—C17—C18	113.8 (3)	N4—C37—C38	115.1 (3)
C17—C18—Cl2	109.4 (3)	C37—C38—Cl4	109.8 (3)
C17—C18—H18A	109.8	C37—C38—H38A	109.7
Cl2—C18—H18A	109.8	Cl4—C38—H38A	109.7
C17—C18—H18B	109.8	C37—C38—H38B	109.7
Cl2—C18—H18B	109.8	Cl4—C38—H38B	109.7
H18A—C18—H18B	108.2	H38A—C38—H38B	108.2
C13—C19—H19A	109.5	C33—C39—H39A	109.5
C13—C19—H19B	109.5	C33—C39—H39B	109.5
H19A—C19—H19B	109.5	H39A—C39—H39B	109.5
C13—C19—H19C	109.5	C33—C39—H39C	109.5
H19A—C19—H19C	109.5	H39A—C39—H39C	109.5
H19B—C19—H19C	109.5	H39B—C39—H39C	109.5
C15—C20—H20A	109.5	C35—C40—H40A	109.5
C15—C20—H20B	109.5	C35—C40—H40B	109.5
H20A—C20—H20B	109.5	H40A—C40—H40B	109.5
C15—C20—H20C	109.5	C35—C40—H40C	109.5
H20A—C20—H20C	109.5	H40A—C40—H40C	109.5
H20B—C20—H20C	109.5	H40B—C40—H40C	109.5
			
C7—N1—C1—C6	5.1 (8)	C27—N3—C21—C26	−2.7 (8)
C7—N1—C1—C2	−178.9 (4)	C27—N3—C21—C22	175.1 (4)
C6—C1—C2—C3	−1.4 (7)	C26—C21—C22—C23	−1.0 (7)
N1—C1—C2—C3	−177.6 (4)	N3—C21—C22—C23	−178.9 (4)
C1—C2—C3—C4	1.7 (7)	C21—C22—C23—C24	1.4 (7)
C1—C2—C3—C9	−179.1 (5)	C21—C22—C23—C29	−178.7 (5)
C2—C3—C4—C5	−2.7 (8)	C22—C23—C24—C25	−0.9 (8)
C9—C3—C4—C5	178.2 (5)	C29—C23—C24—C25	179.1 (5)
C3—C4—C5—C6	3.2 (8)	C23—C24—C25—C26	0.1 (8)
C3—C4—C5—C10	−177.4 (5)	C23—C24—C25—C30	179.9 (5)
C2—C1—C6—C5	1.9 (7)	C24—C25—C26—C21	0.3 (7)
N1—C1—C6—C5	177.7 (4)	C30—C25—C26—C21	−179.6 (5)
C4—C5—C6—C1	−2.7 (7)	C22—C21—C26—C25	0.1 (7)
C10—C5—C6—C1	177.8 (5)	N3—C21—C26—C25	177.9 (5)
C1—N1—C7—O1	−0.7 (7)	C21—N3—C27—O3	−1.1 (7)
C1—N1—C7—C8	178.9 (5)	C21—N3—C27—C28	177.6 (5)
O1—C7—C8—Cl1	77.7 (4)	O3—C27—C28—Cl3	−74.7 (5)
N1—C7—C8—Cl1	−102.0 (3)	N3—C27—C28—Cl3	106.6 (4)
C17—N2—C11—C16	6.3 (7)	C37—N4—C31—C36	−0.8 (8)
C17—N2—C11—C12	−177.3 (4)	C37—N4—C31—C32	−175.9 (4)
C16—C11—C12—C13	−1.7 (7)	C36—C31—C32—C33	2.7 (7)
N2—C11—C12—C13	−178.2 (4)	N4—C31—C32—C33	178.2 (4)
C11—C12—C13—C14	1.5 (7)	C31—C32—C33—C34	−1.9 (7)
C11—C12—C13—C19	−179.6 (5)	C31—C32—C33—C39	179.1 (5)
C12—C13—C14—C15	−1.6 (8)	C32—C33—C34—C35	0.6 (8)
C19—C13—C14—C15	179.5 (5)	C39—C33—C34—C35	179.7 (4)
C13—C14—C15—C16	2.0 (8)	C33—C34—C35—C36	−0.3 (8)
C13—C14—C15—C20	−178.4 (5)	C33—C34—C35—C40	−179.6 (4)
C12—C11—C16—C15	2.0 (7)	C34—C35—C36—C31	1.1 (7)
N2—C11—C16—C15	178.2 (4)	C40—C35—C36—C31	−179.6 (5)
C14—C15—C16—C11	−2.2 (7)	C32—C31—C36—C35	−2.3 (7)
C20—C15—C16—C11	178.2 (5)	N4—C31—C36—C35	−177.3 (4)
C11—N2—C17—O2	−0.7 (7)	C31—N4—C37—O4	2.2 (7)
C11—N2—C17—C18	179.7 (4)	C31—N4—C37—C38	−177.3 (5)
O2—C17—C18—Cl2	73.2 (4)	O4—C37—C38—Cl4	72.7 (5)
N2—C17—C18—Cl2	−107.1 (3)	N4—C37—C38—Cl4	−107.9 (3)

### Hydrogen-bond geometry (Å, °)

**Table:**

*D*—H···*A*	*D*—H	H···*A*	*D*···*A*	*D*—H···*A*
N1—H1N···O4^i^	0.86	2.14	2.983 (4)	168
N2—H2N···O3^ii^	0.86	2.12	2.975 (4)	171
N3—H3N···O2^iii^	0.86	2.15	3.000 (4)	170
N4—H4N···O1^iv^	0.86	2.13	2.987 (4)	172

Symmetry codes: (i) −*x*, −*y*+1, *z*+1/2; (ii) −*x*+1/2, *y*+1/2, *z*+1/2; (iii) −*x*+1/2, *y*+1/2, *z*−1/2; (iv) −*x*, −*y*, *z*−1/2.
